# A Web-Delivered Acceptance and Commitment Therapy Intervention With Email Reminders to Enhance Subjective Well-Being and Encourage Engagement With Lifestyle Behavior Change in Health Care Staff: Randomized Cluster Feasibility Stud

**DOI:** 10.2196/18586

**Published:** 2020-08-07

**Authors:** Menna Brown, Nic Hooper, Phillip James, Darren Scott, Owen Bodger, Ann John

**Affiliations:** 1 Swansea University Medical School Swansea University Swansea United Kingdom; 2 Department of Health and Social Sciences University of the West of England Bristol United Kingdom; 3 Department of Computer Science Swansea University Swansea United Kingdom

**Keywords:** well-being, mental health, behavior change, acceptance and commitment therapy, web-delivered intervention, gamification, adherence and engagement, internet-based intervention

## Abstract

**Background:**

Poor mental health and emotional well-being can negatively impact ability to engage in healthy lifestyle behavior change. Health care staff have higher rates of sickness and absence than other public sector staff, which has implications at both individual and societal levels. Individual efforts to self-manage health and well-being which add to the UK mental health prevention agenda need to be supported.

**Objective:**

The objective of this study was to establish the feasibility and acceptability of the inclusion of a self-guided, automated, web-based acceptance and commitment therapy intervention in an existing health promotion program, to improve subjective well-being and encourage engagement with lifestyle behavior change.

**Methods:**

For this 12-week, 4-armed, randomized controlled cluster feasibility study, we recruited participants offline and randomly allocated them to 1 of 3 intervention arms or control (no well-being intervention) using an automated web-based allocation procedure. Eligibility criteria were current health care staff in 1 Welsh health board, age≥18 years, ability to read English, and ability to provide consent. The primary researcher was blinded to cluster allocation. Feasibility outcomes were randomization procedure, acceptance of intervention, and adherence to and engagement with the wider program. We evaluated health and well-being data via self-assessment at 2 time points, registration and postintervention, using the 14-item Warwick-Edinburgh Mental Well-Being Scale, the 4-item Patient Health Questionnaire, and the 7-item Acceptance and Action Questionnaire—Revised.

**Results:**

Of 124 participants who provided consent and were randomly allocated, 103 completed full registration and engaged with the program. Most participants (76/103) enrolled in at least one health behavior change module, and 43% (41/96) of those randomly allocated to an intervention arm enrolled in the well-being module. Adherence and engagement was low (7/103, 6.8%), but qualitative feedback was positive.

**Conclusions:**

The procedure and randomization process proved feasible, and the addition of the well-being module proved acceptable to health care staff. However, participant engagement was limited, and no one completed the full 12-week program. User feedback should be used to develop the intervention to address poor engagement. Effectiveness should then be evaluated in a full-scale randomized controlled trial, which would be feasible with additional recruitment.

**Trial Registration:**

International Standard Randomised Controlled Trial Number (ISRCTN) 50074817; http://www.isrctn.com/ISRCTN50074817

## Introduction

### Background

Poor mental health and emotional well-being underpin many physical diseases and unhealthy lifestyles and can negatively impact an individual’s ability to engage in healthy lifestyle behavior change [1-3]. Recent recognition that positive mental health and emotional well-being are fundamental components of good health has given rise to a strong UK mental health prevention agenda. For example, the *Five Year Forward view for Mental Health* [4], the *Prevention Concordat for Better Mental Health* [5] and the National Health Service (NHS) *Long Term Plan* [6] saw local and national services come together (in 2019) at an unprecedented level in their commitment to addressing the mental health crisis. This development built on earlier publications from the UK Department of Health and Social Care, which outlined the importance of well-being and its role in health outcomes [7-9]. Economic analysis has further supported the case for greater investment in mental well-being [10]. This recent and sustained UK focus on well-being builds on global recognition that mental health and emotional well-being are a fundamental component of good health [11].

### Mental Health: United Kingdom Picture

Staff sickness and absenteeism in the public sector is high, more so than that of the private sector [12]. Stress is the most commonly cited reason for absenteeism [13], and the associated economic cost is significant at an estimated £105.2 billion a year [14].

Further to their own mental health and well-being needs, health care staff are well placed to promote positive lifestyle behaviors to others, through effective role modelling [15]. Personal health behaviors are critical in establishing effective and confident health behaviors [16,17]. Frontline staff have daily contact with patients and the public and can exhibit health behaviors to be emulated by others. In the United Kingdom, the Nursing and Midwifery Council identified role modelling as a statutory requirement, stating that nurses must “take every opportunity to encourage health-promoting behaviour through education, role modelling and effective communication” [18]. Likewise, for UK medical professionals, the General Medical Council include clear expectations in *Outcomes for Graduates* (pg 23) [19]. Research findings have highlighted the need for ongoing support for health care workers to improve their own health and to fully realize their potential as credible role models and healthy-living advocates for the populations they serve [20-24]. Indeed, Public Health England recently launched Every Mind Matters [25], a website to support well-being and physical health. The proliferation of interest in this area serves to highlight the need for formal evaluation of these approaches.

### Web-Based Approach

Global mental health prevention has incorporated diverse initiatives directed at different levels in society, such as individual, community, and societal level regulations. One key area, which has seen exponential growth, is that of web-delivered interventions. The cost-effective benefits of evidence-based, web-delivered therapies that improve mental health are well established [26-28]; however, poor adherence and engagement remain a significant factor that limits effectiveness [29,30] and application.

To address poor adherence and engagement, this study used a therapeutic approach associated with positive adherence: acceptance and commitment therapy (ACT) [31,32]. ACT is based on the principle of psychological flexibility, which involves accepting one’s unwanted thoughts and feelings while moving toward personal values. Several successful web-based ACT intervention studies have been reported [33-40], for example, in the treatment of depression among smokers [37] and in the prevention of mental health problems among university students [38]. Earlier systematic reviews and meta-analyses have found web-delivered ACT to be effective for the management of depression [41], and others report its effectiveness in both group and individual settings [42-45].

### Randomized Controlled Feasibility Study

This study will provide initial insight into the impact, acceptance, and feasibility of a web-delivered, multifaceted workplace lifestyle behavior change program, which incorporates an ACT-based well-being intervention to support staff mental health in the context of a preventive approach.

### Objectives

The study objectives were to (1) determine whether the randomization procedure was feasible, (2) determine whether the inclusion of an ACT-based well-being intervention, within a web-based lifestyle behavior change program, was acceptable to health care staff, (3) determine whether the well-being intervention positively affected adherence and engagement to the wider program, and (4) explore the impact of additional intervention elements.

## Methods

### Trial Design

This was a 4-armed, cluster randomized controlled feasibility trial.

Trial consent, registration, and assignment to trial arm were automated. The principal researcher (MB) was blinded to cluster allocation throughout the trial; participants were not. A computer code written in Python randomly allocated each cluster to a trial arm using a built-in randomization function. No changes to the program were made during the trial period.

### Trial Arms

The control group used Champions for Health, which consisted of 5 lifestyle behavior change modules: Quit Smoking, Drink Responsibly, Weight Optimization, Regular Exercise, and Eat Healthily.

Intervention 1 group used Champions for Health plus the ACT-based well-being module (ACTivate your Well-being).

Intervention 2 group used Champions for Health and ACTivate your Well-being, plus 5 premade well-being films (see Multimedia Appendix 1).

Intervention 3 group used Champions for Health and ACTivate your Well-being, plus a static social norm message (eg, “Other users like you have reduced their weight, on average to 75 kg.”).

### Ethics

The study received ethical approval from the College of the Human and Health Sciences and the College of Medicine Research Ethics Committee, Swansea University, Swansea, UK, and research and development approval from Abertawe Bro Morgannwg University Health Board Joint Study Review Committee 2017 as service evaluation. The trial is registered with the ISRCTN registry (50074817; Multimedia Appendix 2 [46])

### Clusters

Staff from 1 health board in Wales, UK participated. A health board is an organizational and administrative unit consisting of hospitals, community clinics, and general practices (primary care). There are 7 in Wales.

We created 4 clusters based on key hospital and community sites within the participating health board: 3 clusters received the intervention and 1 did not (the control). Use of this trial design is common in health care contexts where cluster trials are an important methodology used to compare different ways of encouraging health behavior change [47,48].

Clusters. We selected this design for pragmatic reasons. The reasons were 3-fold. First, focus group discussions identified that participants who had taken part in earlier releases of the website had discussed its content with colleagues. As such it became evident that if we allocated participants at the individual level, they may discuss and share the content of the intervention with those not allocated to that trial arm. This approach is reported elsewhere [49,50]. Second, the clusters are natural groups of people, determined by their place of employment. Outcomes within naturally occurring clusters may tend to be more correlated than those across clusters; this is because individuals within a health board may have similar practices, arising from organizational culture and shared environment or demographic features that might influence the outcome [48]. Third, allocation by site location may support recruitment. Undertaking randomization after consent and baseline would introduce significant delay, which might have a negative effect on enrollment and engagement.

### The Program: Champions for Health

Champions for Health, developed by Public Health Wales, comprised 5 lifestyle behavior change modules. Each included text and images and they were based on the health belief model [51], the theory of planned behavior [52], plan, do, study, act [53], and the self-regulatory model [54].

### The Intervention: ACTivate your Well-being

Following a participatory design process [55-58], NHS staff (n=39), researchers, ACT practitioners, mental health experts, and computer scientists worked together to co-design the website and intervention through a series of exploratory interviews, focus groups, usability sessions, and a pilot evaluation.

We developed an automated, interactive, 12-week, self-guided intervention called ACTivate your Well-being for predetermined sequential release. Recommended time spent on each week was 20 minutes per day, 3 days a week. In addition to the structured modules, 3 pop-ups were available: Green Space gallery, Sleep, and Relaxation. These could be accessed freely.

### Recruitment and Eligibility

All staff employed by the health board at the time of the study were eligible to participate. Eligibility criteria were current health care staff in 1 Welsh health board, age≥18 years, ability to read English, and ability to provide consent.

We recruited participants between January 28 and February 7, 2019 at 4 hospital sites. Electronic invitation and advertisements were displayed on the hospital intranet, electronic, and physical notice boards. Presentations were made at 4 site locations during induction training to a voluntary staff Well-Being Champions scheme.

### Procedure

Participants registered individually via the website. All participants were required to provide consent using a checkbox process prior to completing a registration form, which asked for username, password, gender, age, location, self-rated health, self-rated work performance, sickness leave in the past 6 months, quantity of 1-week absences in the past 30 days, and self-assessed primary outcome measures. Users could opt to receive a semiautomated weekly email reminder, which included the website link and a motivational quote.

Once registered, participants accessed the website freely by logging in to their account. At this point, participants found out whether they had access to the well-being intervention. A personalized dashboard enabled participants to enroll in 1 or more modules and track their progress. We incorporated 2 gamification features to support sustained engagement [59]: Rewards and Feedback. Gamification is the use of game elements in non–game contexts [60]. Health points were rewarded for website engagement and converted into trophies at predetermined thresholds, and a bar showed progress toward each trophy. Feedback graphs were generated to show progress (Multimedia Appendix 3). In week 12 participants were reminded, via the home page and weekly email, to complete the time 2 outcome measures and provide feedback. At this point they were also invited to take part in a focus group. We sent 3 email reminders.

### Outcome Measures

#### Primary Outcome Measure

The 14-item Warwick-Edinburgh Mental Well-Being Scale (WEMWBS) is a validated measure of mental well-being in the general population, responsive to change at both the individual and group levels [61-63]. A higher score represents more positive well-being. A score of 43.5 or less is considered a screening threshold for depression [62]. An increase of 2.77 or greater indicates statistically significant improvement [63].

The 4-item Patient Health Questionnaire (PHQ-4) Anxiety and Depression Scale [64] screened for anxiety and depression. On each subscale, a score of 3 (range 0-6) or greater is considered positive for screening purposes for anxiety and depression [64].

#### Process Measures

The 7-item Acceptance and Action Questionnaire—Revised (AAQ-II) is a validated, 1-factor measure of psychological inflexibility [65]. Higher scores (range 7-49) indicate greater levels of psychological inflexibility [65]. Cutoff points are not published for this measurement tool; however, a score of 17.5 and greater has been identified to indicate significant psychological inflexibility [66].

### Sample Size Considerations

Feasibility trial designs do not commonly employ formal power calculations [67]. As such we aimed to recruit 100 participants, 25 in each trial arm, to allow comparison across groups and to explore the study objectives.

### Data Analysis

#### Randomization Procedure

Functionality was assessed by the web developer during week 1 to ensure that self-reported location data were used accurately to populate trial arms.

#### Acceptability, Adherence, and Engagement

Descriptive statistics reported website registration and participant characteristics. We conducted statistical analysis using nonparametric methods in IBM SPSS version 26 (IBM Corporation) to explore baseline characteristics of each trial arm. Adherence was measured by completion of outcome measure at baseline and postintervention. Qualitative feedback, collected at the end of the program via a structured feedback form, interview, and focus group discussion, was audio recorded and transcribed verbatim. Data were analyzed using inductive thematic analysis [68].

#### Primary Outcome Effect

The feasibility study was not powered to test statistical significance, but merely to explore the impact and effectiveness of the intervention on lifestyle behavior change and well-being across trial arms.

## Results

### Randomization

The automated randomization procedure proved effective, and participants were allocated as expected.

### Recruitment and Participant Summary

**Figure 1:**
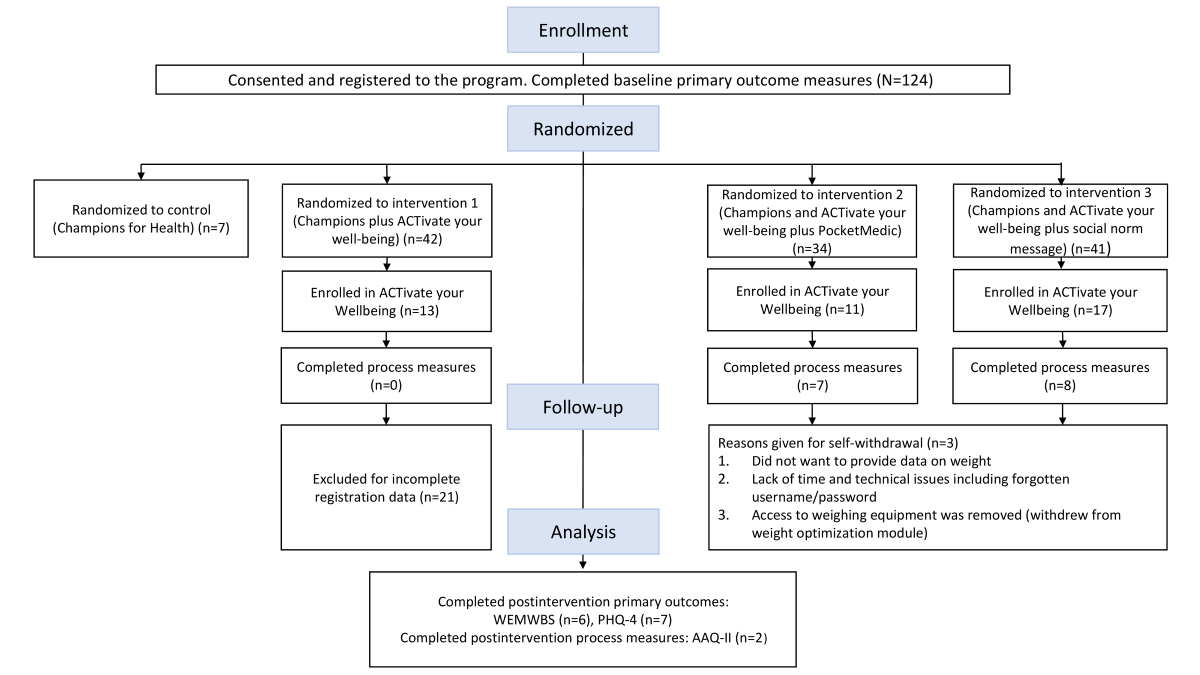
Consolidated Standards of Reporting Trials flow diagram. AAQ-II: Acceptance and Action Questionnaire—Revised; PHQ-4: Patient Health Questionnaire-4; WEMWBS: Warwick-Edinburgh Mental Well-Being Scale.

A total of 124 users consented to participate and were randomly allocated to a trial arm. Of these, 103 users provided baseline data for the primary outcome measures and were analyzed (Figure 1).

Users spanned all age brackets (18-65 years old). Most users (91/103, 88.4%) were female and rated their health as “good” to “excellent” (82/101, 81.2%) on a 5-point Likert scale. Over half (63/103, 61.2%) reported no days off work in the past 6 months and, in line with this, the majority (81/103, 78.6%) did not report any 1-week absences in the past month. On average, users rated their general work performance 8 on a scale of 1 to 10, with 10 being highest. All opted to receive email reminders (Table 1).

**Table 1:** Participant characteristics (n=103).

Characteristics		Trial arm			
		Control (Champions for Health)	Intervention 1 (Champions plus ACTivate your Well-being^a^)	Intervention 2 (Champions and ACTivate plus PocketMedic)	Intervention 3 (Champions and ACTivate your Well-being plus social norm message)
Randomized, n		7	42	34	41
Analyzed, n		7	21^b^	34	41
Female, n (%)		6 (86)	19 (90)	30 (88)	38 (93)
**Age bracket (years), n (%)**					
	18-25	0	0	4 (12)	2 (5)
	26-35	2 (28)	8 (38)	10 (29)	10 (24)
	36-45	3 (43)	5 (24)	11 (32)	12 (29)
	46-55	1(14)	5 (24)	7 (20)	14 (32)
	56-65	1 (14)	3 (14)	2 (6)	4 (10)
Self-reported days off work, mean (SD)		1.4 (2.1)	9.3 (18.4)	1.0 (1.5)	6.9 (25.3)
Self-rated general work performance score^c^, mean (SD)		7.3 (1.6)	7.6 (1.7)	7.5 (1.3)	7.5 (1.7)

^a^ACTivate your Well-being intervention based on acceptance and commitment therapy.

^b^21 users were excluded based on incomplete registration.

^c^On a scale of 1-10, with 10 being the highest.

### Trial Arms

We detected no significant differences between trial arms, using the Kruskal-Wallis test, at baseline (n=103) for registration week (*P*=.17), age (*P*=.51), self-rated health (*P*=.36), days off work (*P*=.84), absences of 1-week duration (*P*=.09), self-rated work performance (*P*=.94), WEMWBS (*P*=.24), PHQ-4 (*P*=.27), AAQ-II (*P*=.25) or gender (χ^2^_3_=103; *P*=.56).

We observed no difference for enrollment in module: Weight Optimization, χ^2^_2_=103, *P*=.38; Regular Exercise, χ^2^_2_=103, *P*=.25; Drink Responsibly, χ^2^_3_=103, *P*=.88; Eat Healthily, χ^2^_3_=103, *P*=.74; Quit Smoking, χ^2^_3_=103, *P*=.85). However, module engagement differed across intervention groups (χ^2^_2_=96, *P*=.40).

Enrollment (χ^2^_2_=96, *P*=.10) or engagement (as a binary variable) in the well-being intervention did not differ significantly between the 3 intervention groups (χ^2^_2_=96, *P*=.79) (Table 2).

**Table 2:** Module engagement (no. of participants) per trial arm (n=103).

Module			Trial arm						
			Control (Champions for Health)		Intervention 1 (Champions and ACTivate your Well-being^a^)		Intervention 2 (Champions and ACTivate your Well-being plus PocketMedic)		Intervention 3 (Champions and ACTivate your Well-being plus social norm message)
Randomized, n			7		21		34		41
**Enrolled, n (%)**									
	Champions for Health	5 (71)		13 (62)		29 (85)		27 (66)	
	ACTivate your Well-being	N/A^b^		13 (62)		11 (32)		17 (41)	
**Engaged, n (%)**									
	Champions for Health	2 (28)		0		9 (26)		9 (22)	
	ACTivate your Well-being	N/A		1 (5)		3 (9)		4 (10)	

^a^ACTivate your Well-being intervention based on acceptance and commitment therapy.

### Outcome Measures

#### Primary Outcome Measure

At baseline, participant well-being scores, measured using WEMWBS, was mean 46.3 (SD 8.8, range 29-68). This was lower than the general population. Only 6 participants completed WEMWBS postintervention, and this group recorded a higher score (mean 53.8, SD 3.1) and higher minimum score (43). When this subgroup was tested, 5 participants showed raised scores, which presented some evidence of an improvement, but this fell below the level of statistical significance (*P*=.11; Wilcoxon signed rank test).

Baseline anxiety and depression scores, measured using PHQ-4, were within the normal population range, with means of 1.8 (SD 1.6) and 1.3 (SD 1.5), respectively. A small subset of users met the screening criteria (20/103, 19.4%; 11/103, 10.7%, respectively). The combined PHQ-4 scores (n=7) did not change significantly postintervention (*P*=.34; Wilcoxon signed rank test).

#### Process Measures

The mean AAQ-II score was 21.7 (SD 9.8; n=22). However, no comparison could be drawn, as only 2 participants completed the postintervention questionnaire.

### Acceptability, Adherence, and Engagement

Adherence to the study protocol was poor (7/103, 6.8%). However, the majority of participants (76/103, 73.8%) enrolled in at least one module. Almost half (50/103, 48.5%) enrolled in 1 module, 17 (16.5%) enrolled in 2 modules, 7 (6.8%) enrolled in 3 modules, and 2 (1.9%) enrolled in 4 modules. The most popular modules were well-being (41/96, 43%), Regular Exercise (40/103, 38.8%), and Weight Optimization (39/103, 37.9%).

Of the 9 participants who enrolled in Drink Responsibly, only 3 (33%) engaged. However, health outcomes improved for these active users; 1 reported a reduction in days per week that they consumed alcohol from 4 to 2, with a reduction from 20 drinks per week to 8. Another reduced their consumption from 4 to 3 days per week, with a reduction from 11 to 5 drinks, and the third increased their number of days drinking but their overall alcohol consumption reduced (from 18 to 4). Of the 23 participants (22.3%) who enrolled in Eat Healthily, only 6 (26%) engaged, 1 until week 6. User data indicated poor fruit and vegetable consumption, with few meeting recommended guidelines and some never consuming the recommended 5-a-day portion (5/23). Almost all who enrolled in Weight Optimization (37/39, 95%) engaged and provided an initial weight (mean 75.77, SD 15.80 kg). A small subsample (10/39, 26%) provided a second weight (mean 75, SD 10.89 kg). Of the 40 participants who enrolled in Regular Exercise, 33% (13/40) actively engaged. This module saw the longest sustained engagement of the modules, with activity recorded until week 9. Only 2 (2/103, 1.9%) participants enrolled in Quit Smoking and neither entered nor tracked any data. Well-being had the highest enrollment (41/96, 42%). However, of those who enrolled, only 7 (17%) completed any of the weekly “try-now” activities. This module had the longest sustained engagement overall, with 1 user active until week 10.

### Qualitative Data

To fully explore acceptability, we collected a range of qualitative data. We facilitated 2 one-to-one interviews (25-minute duration) and 2 focus groups (77- and 73-minute durations, 4 participants each). A total of 15 participants contacted the principal researcher via email and 8 completed the feedback survey.

Qualitative feedback was positive (Table 3), and it was clear that staff welcomed the inclusion of the well-being intervention; indeed, people who had used the prior releases requested its inclusion. Analysis identified recommendations for future development.

**Table 3:** Sample quotes from participants’ feedback and their recommendations.

Theme	Illustrative quotes and recommendations
Feedback on website and well-being module	“I liked that it would tell you what you should be doing to keep up with the NHS^a^ recommendations and then how it compared that to Wales and the rest of the population. I liked all that information.” [Interview]
	“I would have liked to set weekly goals.” [Interview]
	“Easy to use, visually was nice.” [Interview]
	“The health and fitness aspects were quite helpful, I was enrolled on the modules, health eating, weight management, and regular exercise. They were quite simple and straightforward and the information there was very useful.” [Interview]
	“I really liked the PocketMedic. I looked at all the films on there.” [Focus group]
	“I liked some aspects of the ACT^b^ therapy and I found some aspects helpful. Certainly, I’ve suffered from intrusive thoughts and it’s helpful to sort of just accept them rather than fighting against them.” [Interview]
Nonadherence	“To me it was purely entering my weight, which I understand needed to be done...where I sit in my office everyone can see my screen clearly and that was why I was not happy to enter my weight.” [Email]
	“Couldn’t log in, then other priorities took over firefighting through work. It’s a time factor thing.” [Email]
	“I stopped doing the one about weight management, as when I started there were scales up in outpatients that I used to use and then they took them away.” [Interview]
Participant recommendations	Provide an option to set weekly goals and the option to report whether this goal was achieved or not.
	Provide the option to return to the previous week to enter progress data.
	Display progress data per activity undertaken; for example, during week 1 you swam for a total of 80 minutes; you did yoga for 60 minutes, or more detailed track-your-progress options to facilitate competitive and personalized elements.
	Provide personalization of the profile area. Provide the option to edit data displayed on the screen specifically in reference to weight due to lack of privacy in the work setting or the option to hide private details such as weight.
	Streamline access to well-being exercises and activities.
	Include additional signposts to alternative sources of help.
	Incorporate opportunities to connect and interact with others.
	Improve the layout of AAQ-II^c^.

^a^NHS: National Health Service.

^b^ACT: ACTivate your Well-being intervention based on acceptance and commitment therapy.

^c^AAQ-II: Acceptance and Action Questionnaire—Revised.

## Discussion

### Principal Findings

This study explored the feasibility and acceptability of the inclusion of an ACT-based well-being intervention, within an existing web-based, workplace lifestyle behavior change program for health care staff. The cluster design and automated randomization procedure proved feasible. Participants were successfully randomly allocated based on self-reported location, and the principal researcher remained blinded until postintervention. The new multifaceted program also proved acceptable to NHS staff. Recruitment was positive and participation rates compared equally with previous releases; for example, the 2015 campaign administered by Public Health Wales recruited 140 staff from 1 health board.

The proportion of users (43%) who selected to enroll in the well-being intervention highlighted that initiatives such as these are desirable and that well-being support is required in the workplace. Indeed, the qualitative interviews, focus groups, and feedback were positive, and this is encouraging. Staff who engaged with the program and well-being module enjoyed the resources and reported personal benefit. Feedback also highlighted specific ways to develop the intervention to address poor engagement from the staff perspective, which will be used to support future development. However, the lack of engagement is still a concern. Only 7 participants were active in the try-now elements of the intervention, and engagement was equally poor across the modules. It is worth noting that poor engagement may have occurred as an artifact of what was measured. Specifically, the website recorded activity only for “track your progress:” it was not possible to assess log-on rate, time spent on each page, and general website activity. Therefore, it is possible that users engaged with other program elements. Indeed, the focus group and interview data suggested that the site overall was well used. In line with this, it is also encouraging that most participants enrolled in and engaged with at least one module, the most popular being Regular Exercise and Weight Optimization. Indeed, these were often selected in combination. Quit Smoking was the least popular, with only 2 users, despite local rates of smoking remaining at 22% [69].

Looking at the global picture, this study found health care staffs’ well-being scores (WEMWBS) to be lower than the general population (data for England). Mean scores for men and women on this measure are 50.1 and 49.6 [61], respectively, compared with 46.3 in our study. In addition to this, a subset of participants had PHQ-4 scores associated with anxiety and depression. These findings add to the global picture, which suggests prevalence rates of common mental disorders, such as anxiety and depression, are high. In the United Kingdom, estimated population incidence rates are 4% to 10% [70]. Elsewhere, similar instances are reported. For example, lifetime disorder rates in Australia are reported to be 45% [71]. The individual and economic cost associated with common mental disorders is significant. Mental health problems constitute the largest single source of world economic burden, with an estimated global cost of £1.6 trillion [72]. In the United Kingdom, the estimated costs of mental health problems are £70 billion to £100 billion each year and account for 4.5% of gross domestic product [73].

Finally, in this study we were also interested in exploring the impact of the additional intervention elements, PocketMedic and social norm message. There was no significant difference in engagement across the 3 interventions. Qualitative feedback indicated that PocketMedic films were appreciated. Future exploration could collapse the additional elements into one. This is line with earlier findings [74].

### Limitations

Several limitations must be acknowledged.

First, the small sample recruited for this feasibility study limited the statistical analysis undertaken. This was particularly relevant to the control arm, as participants were randomly allocated in a 1:3 ratio in favor of the intervention. While we undertook the same recruitment process at each site, only 7 members of staff registered from the location randomized to the control. However, the sample size is not dissimilar to other published studies reporting the effects of similar interventions [36]. In future, cluster size should be considered.

Second, intervention 1 had a high number of participants excluded from the analysis as a result of incomplete registration data. This further reduced the sample size and equality between trial arms. The registration process should include mandatory fields to avoid this issue in future iterations.

A third study limitation resulted from low adherence. Few participants completed the postintervention outcome measures. This limited exploration of intervention impact. In response it will be important to explore alternative ways to encourage self-reported completion at postintervention. One option is to track individual nonuse and request feedback within 1 week. This may go some way to improving adherence rates, as reasons for nonuse could be identified and resolved during the study period. Alternatively, the intervention could be shortened to support continued use. Another option might be to redesign engagement data collection points within the website. In this version, engagement monitoring was limited to the track-your-progress or try-now features, both of which are user initiated. No automated data were recorded. Feedback and interview data suggested that participants engaged at many additional time points, which was not captured in our analysis. This should be addressed. Equally, the use of rewards and feedback did not support sustained engagement. Future developments should consider use of additional or different gamification features, for example, avatars or social interaction [75], or guided support and structured feedback, which have been associated with better adherence.

Fourth, due to an error in the database, which has been amended, we were not able to examine health care worker role in relation to self-reported absenteeism.

### Comparison With Prior Work

The inclusion of ACTivate your Well-being within the Champions for Health program created a multifaceted program free and easily accessible to a range of health care staff.

Limited research has explored the role of well-being interventions on lifestyle behavior change programs. To our knowledge, this study is one of the first to explicitly explore the additional benefit of an emotional well-being intervention on lifestyle behavior change. We identified 2 prior studies that incorporated a mental health intervention within a physical health promotion program [76,77]; however, neither explicitly evaluated the additional benefit of a well-being intervention on lifestyle behavior choices and neither used ACT.

The web-based interface used in this health care setting offered an opportunity to provide tailored support to public sector staff through convenient and accessible means. The inclusion of the emotional well-being intervention, in combination with the modules, is a significant step forward in terms of prevention and early intervention for self-management of positive health behaviors and builds on the UK mental health prevention agenda. The multifaceted program targeted both physical health behaviors and emotional well-being in 1 integrated program. This is the unique feature of this program.

### Conclusion

This feasibility study was not powered to statistically assess the impact on physical health. A full-scale randomized controlled trial with wider-ranging recruitment methods and additional participant groups would likely support this. We are hopeful the larger-scale trial will answer this question. Study participants who engaged with ACTivate your Well-being and Champions for Health reported positive feedback and made several useful recommendations to take this project forward.

## Appendix

Multimedia Appendix 1 PocketMedic.

Multimedia Appendix 2 CONSORT-EHEALTH checklist V1.6.1.

Multimedia Appendix 3 Champions for Health website images and screenshots.

## Abbreviations

AAQ-II: Acceptance and Action Questionnaire—Revised
ACT: acceptance and commitment therapy
NHS: National Health Service
PHQ-4: 4-item Patient Health Questionnaire
WEMWBS: Warwick-Edinburgh Mental Well-Being Scale
