# Supplementary material for: A Web-Delivered Acceptance and Commitment Therapy Intervention With Email Reminders to Enhance Subjective Well-Being and Encourage Engagement With Lifestyle Behavior Change in Health Care Staff: Randomized Cluster Feasibility Stud
Source: JMIR Form Res. 2020 Aug 7;4(8):e18586. doi: 10.2196/18586 (PMC7442951; doi:10.2196/18586)

www.medic.video/cv-well

The five wellbeing films;

- It could happen to anyone (6:28 minutes)
- Facing up (5:01 minutes)
- The best medicine (6:17 minutes)
- Tried and tested (8:22 minutes)
- Moving on (7:16 minutes)

POCKET MEDIC®

POCKET MEDIC®

Wellbeing

NHS healthcare professionals and patients have worked with the PocketMedic team to make this series of health information films. Each film has been reviewed by clinicians and patients alike to ensure that the information they share can help you to understand and manage your condition. However, if you have any concerns or worries about your health you should contact your healthcare professional for advice.

We would love to know if you found these films helpful. After watching all of the films, please take the time to fill out these four quick questions. Your feedback will help us improve this service. Thank you.
medic.video/feedback-wellbeing

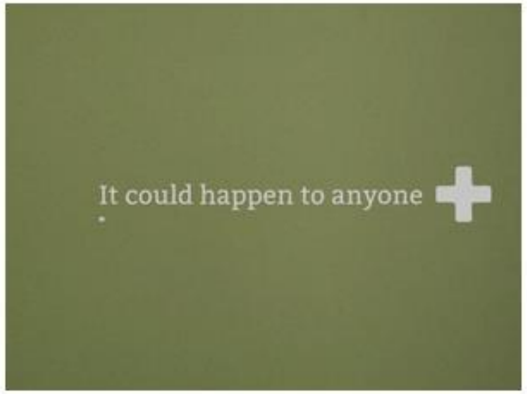

It Could Happen To Anyone

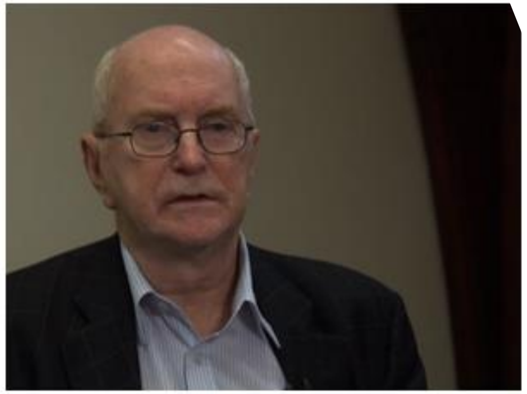

Facing Up

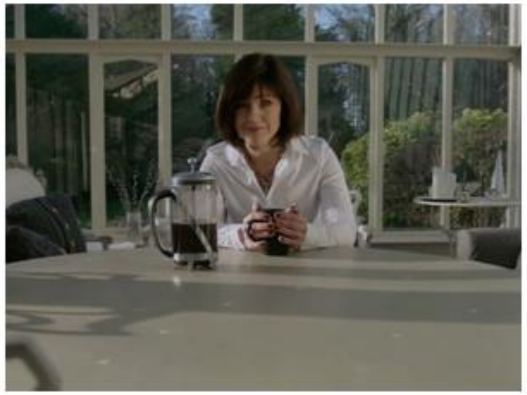

The Best Medicine

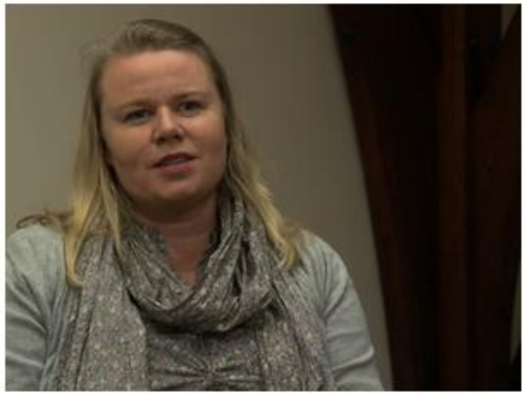

Tried And Tested

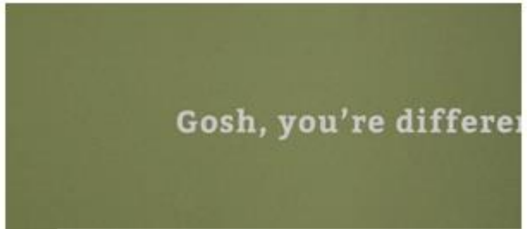

Supplement: Multimedia Appendix 1 [file formative_v4i8e18586_app1.pdf]
