# Supplementary material for: A Web-Delivered Acceptance and Commitment Therapy Intervention With Email Reminders to Enhance Subjective Well-Being and Encourage Engagement With Lifestyle Behavior Change in Health Care Staff: Randomized Cluster Feasibility Stud
Source: JMIR Form Res. 2020 Aug 7;4(8):e18586. doi: 10.2196/18586 (PMC7442951; doi:10.2196/18586)

Champions For Health Home About us Contact us Login Register

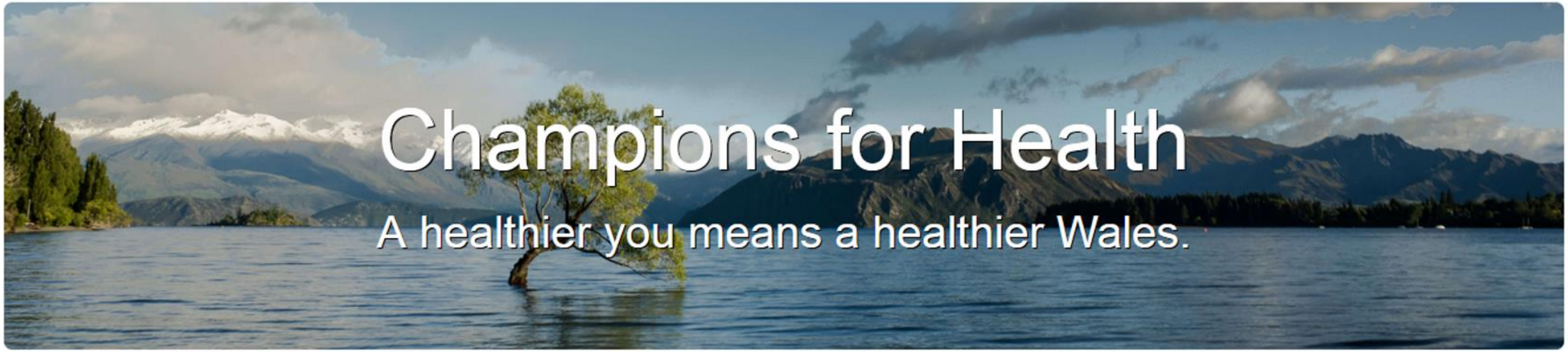

Welcome to Champions for Health: 12 week staff health challenge!

Why take part?

Changing your lifestyle and health choices can be difficult even when you are motivated and aware of the benefits!

Champions is here to help!

Take the 12 week challenge and improve your health and wellbeing alongside your work colleagues.

Your employer has signed up to take part.

How do I take part?

1. Click on the register option on the top right of this page
2. Complete the registration form
3. Select a personal health challenge
4. Visit the website to record and track your progress over the 12 weeks
5. Earn health points and trophies

At the same time, BOOST your wellbeing by trying out the weekly, bite sized interactive exercises and get that positive work life balance back.

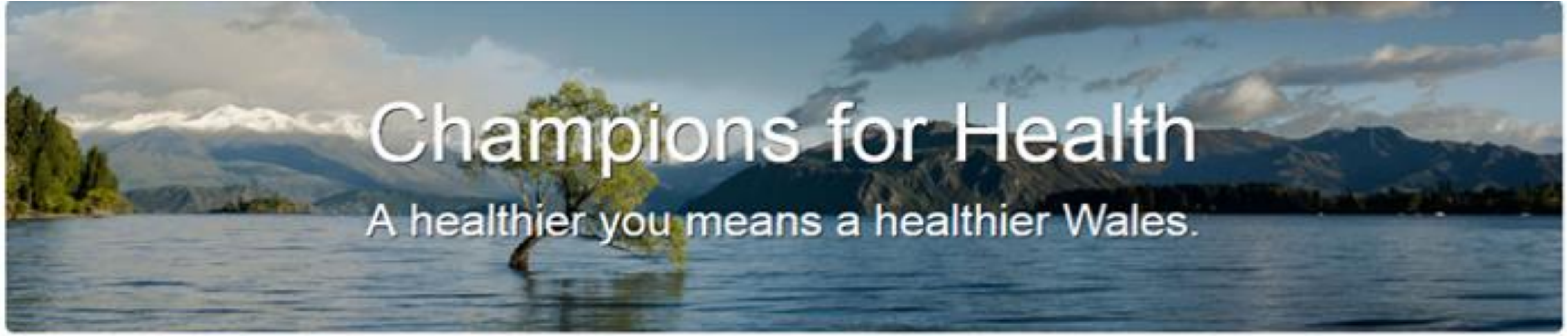

Welcome to Champions for Health, please select your health challenges. Click 'enrol' to enrol onto a new module, or click 'read more' to take part in a module that you have previously enrolled onto.

Quit Smoking

Quitting smoking equals better health. Take this health challenge if you want to stop smoking and beat those cravings. The resources here can support you to reach your goal. Register to find out more.

READ MORE »

Weight Optimisation

Take this health challenge if you are looking to make positive changes to your weight or are wanting that extra bit of motivation to keep going with your healthy weight resolutions. Register to find out more.

READ MORE »

Regular Exercise

Take this health challenge if you want to make a positive change to your health routine. The module includes suggestions to try out, tips and reminders of the all the amazing benefits of an active lifestyle. Register to find out more.

READ MORE »

Eat Healthily

Eating 5-a-day can improve your health! Take this health challenge if you are looking to maximise your intake of fruit and vegetables and boost your overall health. Register to find out more.

ENROL »

Drink Responsibly

Take this health challenge if you want to reduce your alcohol intake and maintain a healthy balance when it comes to those little tipples! Register to find out more.

READ MORE »

Wellbeing module

A collection of techniques and exercises which are known to help support and boost individuals wellbeing.

READ MORE »

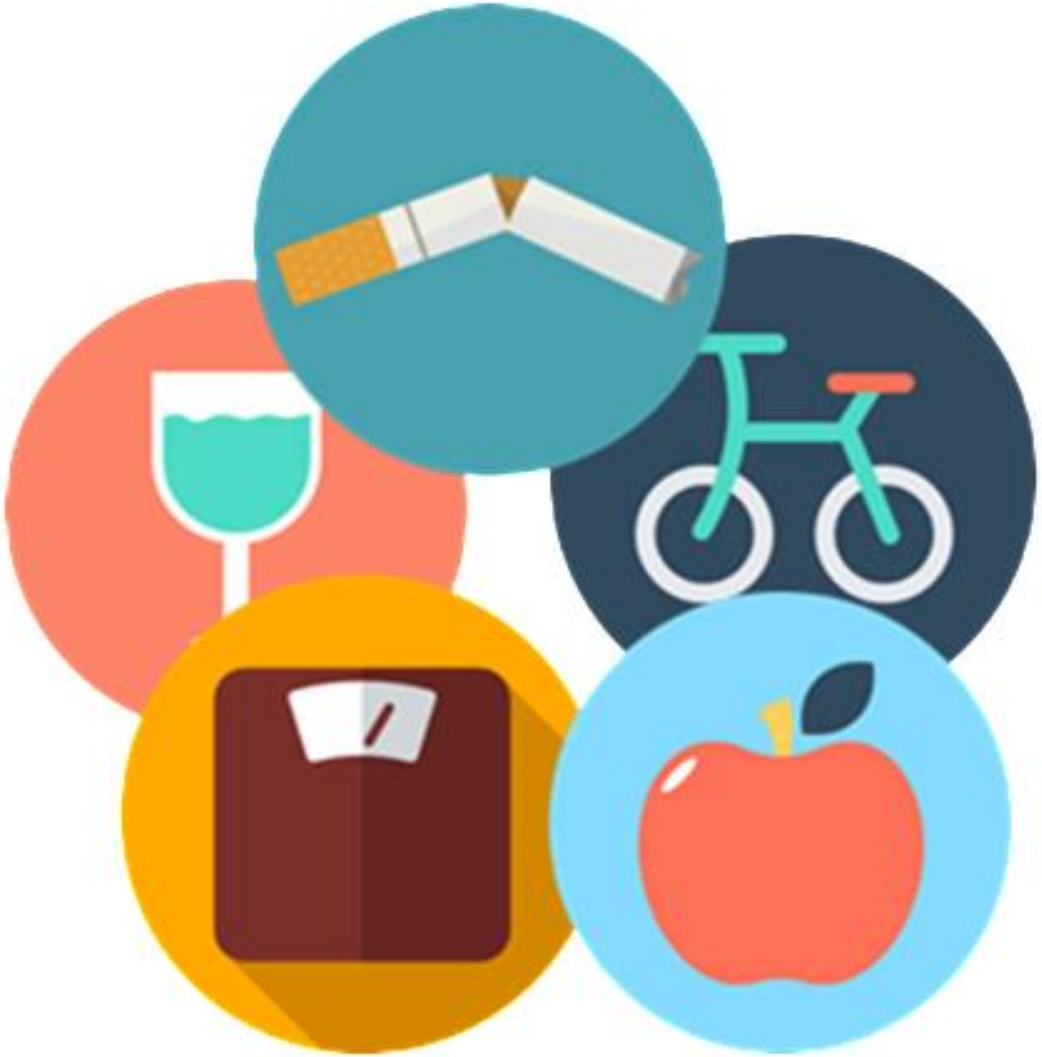

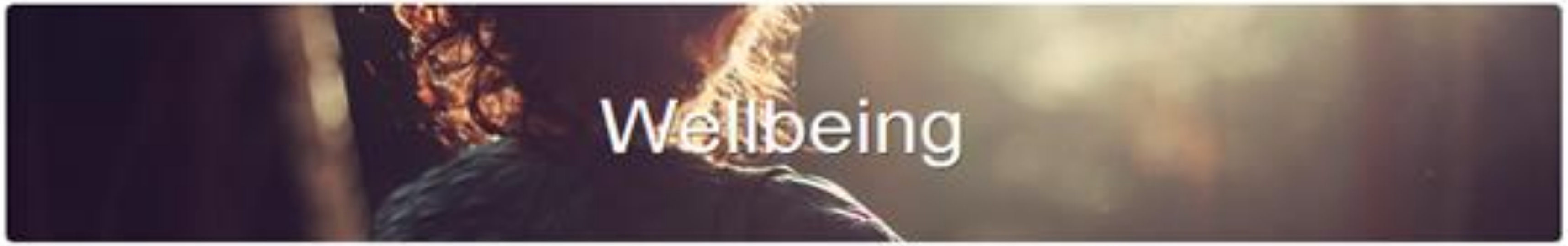

Welcome!

Where to start?

If you're looking for quick fire tips go to Relaxation. If sleep is troubling you look here for suggestions, if you want inspiration look at the Green Space gallery. For a long-term wellbeing boost start with ACT week 1. Here 6 techniques are explained over 12 weeks in bite sized sections. The 'Try Now' activities are quick and easy, the 'Try at Home' exercises can be used when you have more time and get your headphones ready for the 'Watch Now' clips, guided meditations and breathing exercises. Each week you can enter your progress and track your activity over time in your personal dashboard.

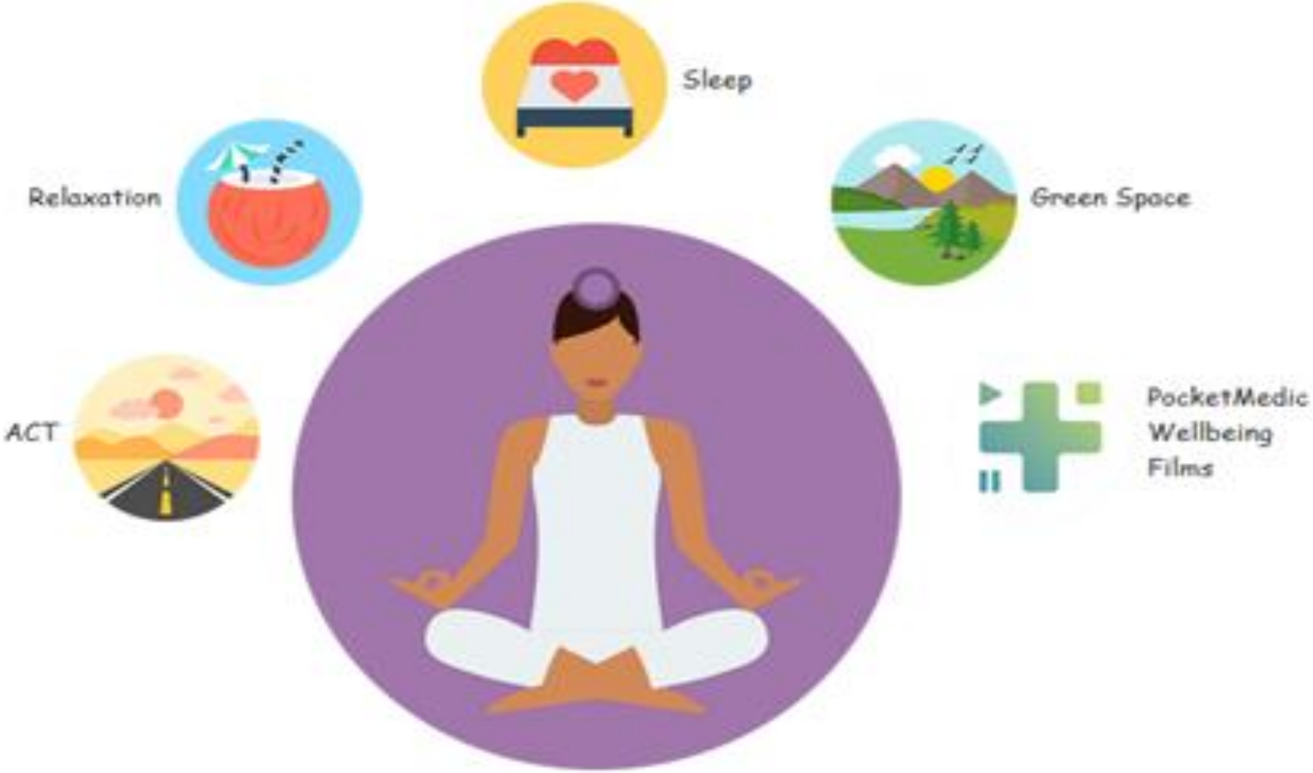

Weekly modules

1. Introduction to ACT and the resource: What is ACT?
2. Personal relevance

1. Values exploration

1. Acceptance

1. Cognitive Defusion

1. Being Present

1. Self as Context

1. Committed Action

1. Psychological Flexibility
2. Barriers
3. Self-Compassion
4. Experiential exercises and additional resources

Optional supplementary modules

1. **Green space gallery**
2. **Relaxation tips and hints**
3. **Psych educational resources on sleep**

User dashboard

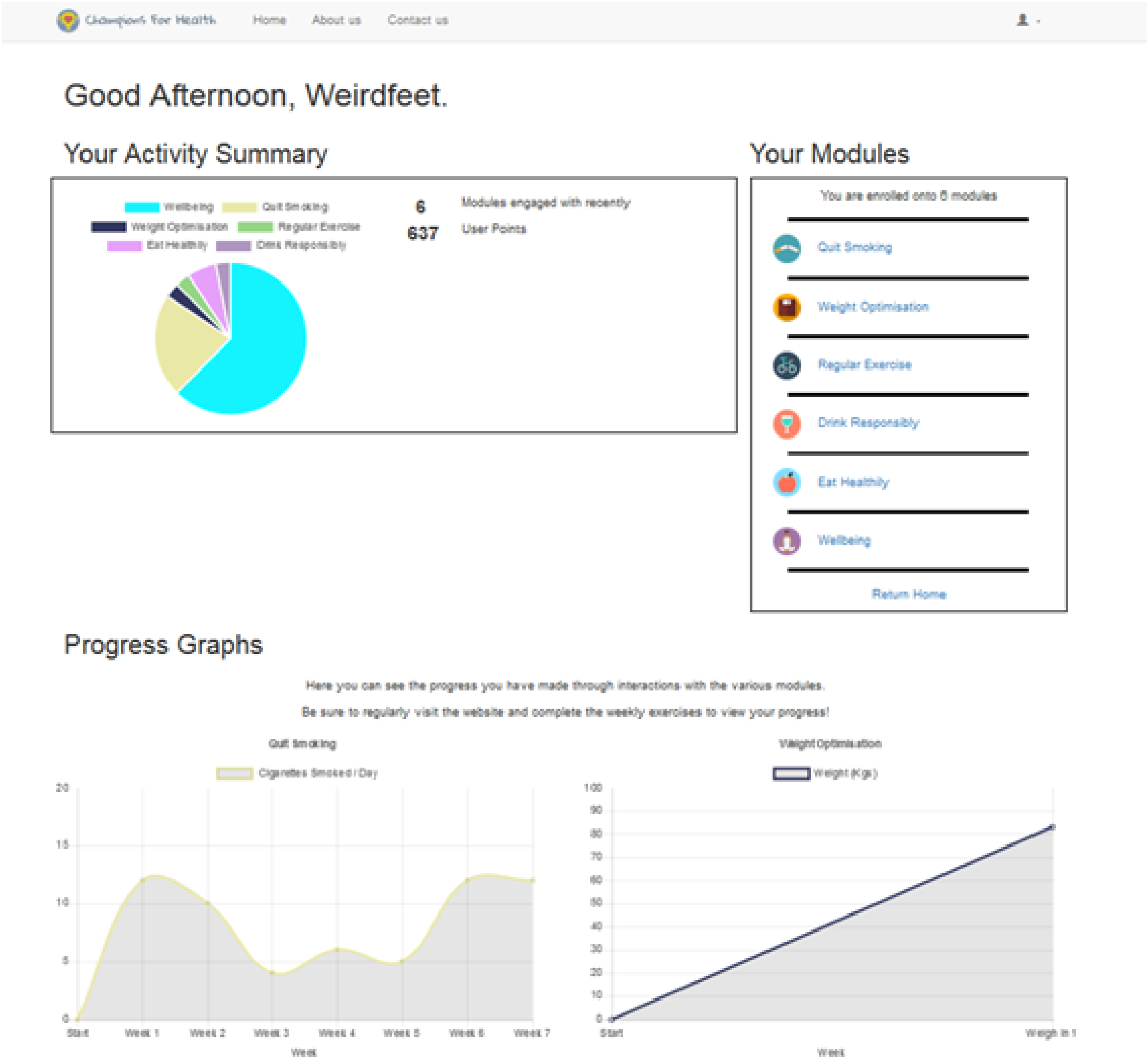

Gamification features

Trophies

You can earn points and trophies by taking part in the activities found in each module and recording your progress. They will appear here in your user dashboard.

You will receive 1 point for each visit and 5 points for each activity you complete. Collect points to unlock new trophies.

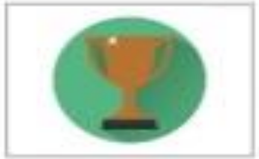

You have earned the Bronze Trophy for achieving 50 points. Congratulations!

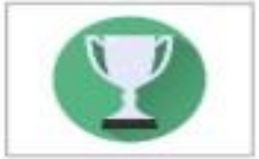

You have earned the Silver Trophy by achieving 100 points. Congratulations!

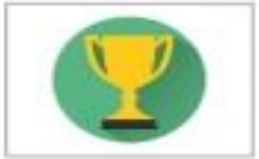

You have earned the Gold Trophy by achieving 200 points. Congratulations!

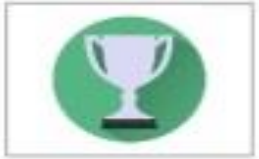

You have earned the Platinum Trophy by achieving 400 points. Congratulations!

Progress to Next Trophy

800/800

Your Activity Summary

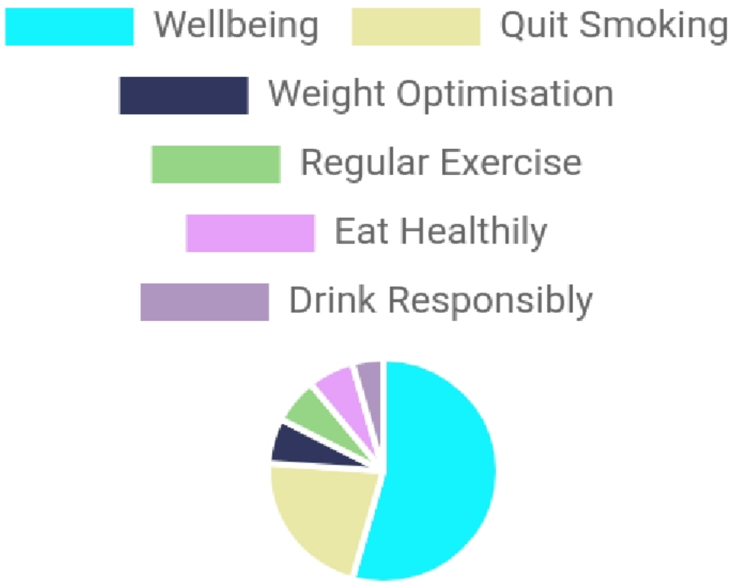

6

Modules engaged with recently

800

User Points

[illegible]

Here you can see the progress you have made through interactions with the various modules.

Be sure to regularly visit the website and complete the weekly exercises to view your progress!

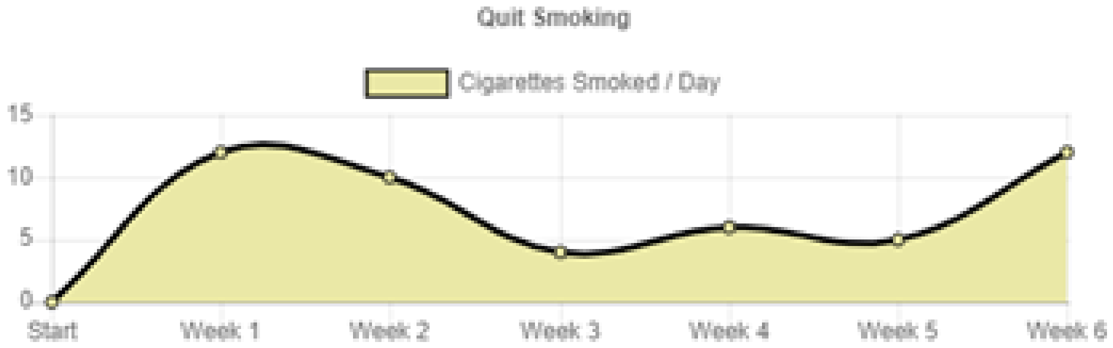

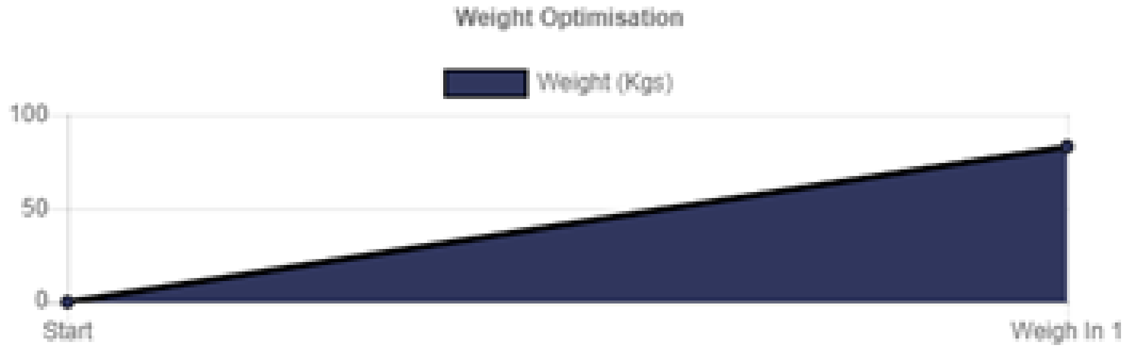

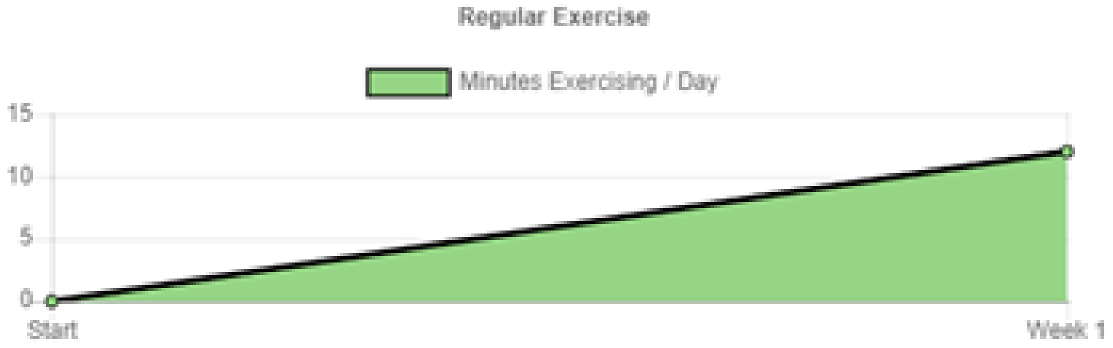

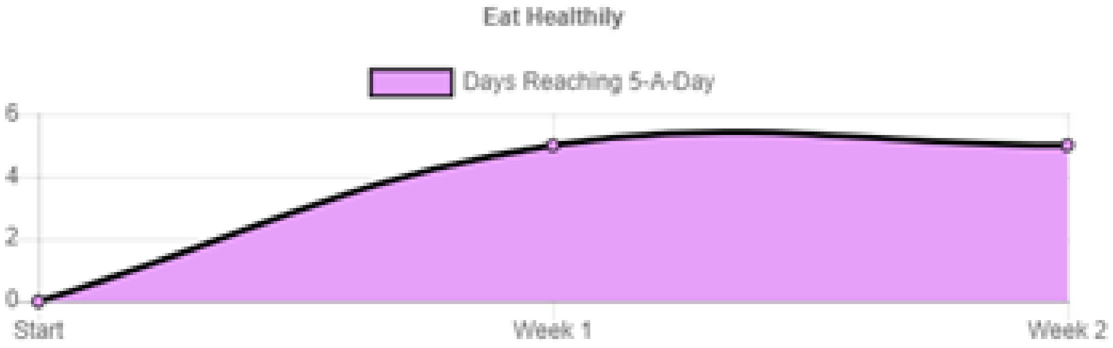

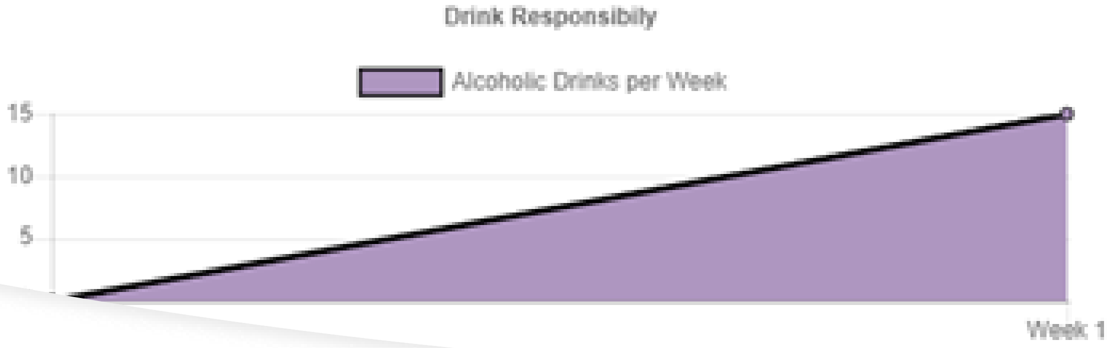

Progress Graphs

Here you can see the progress you have made through interactions with the various modules.

Be sure to regularly visit the website and complete the weekly exercises to view your progress!

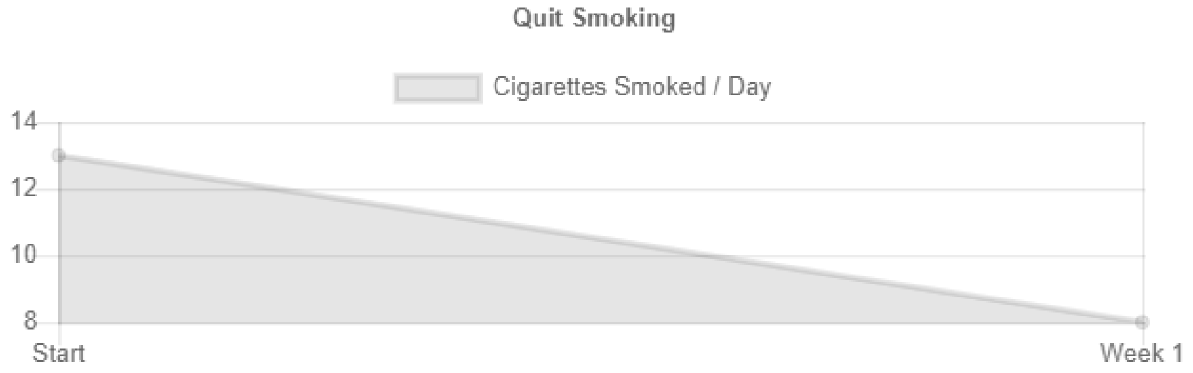

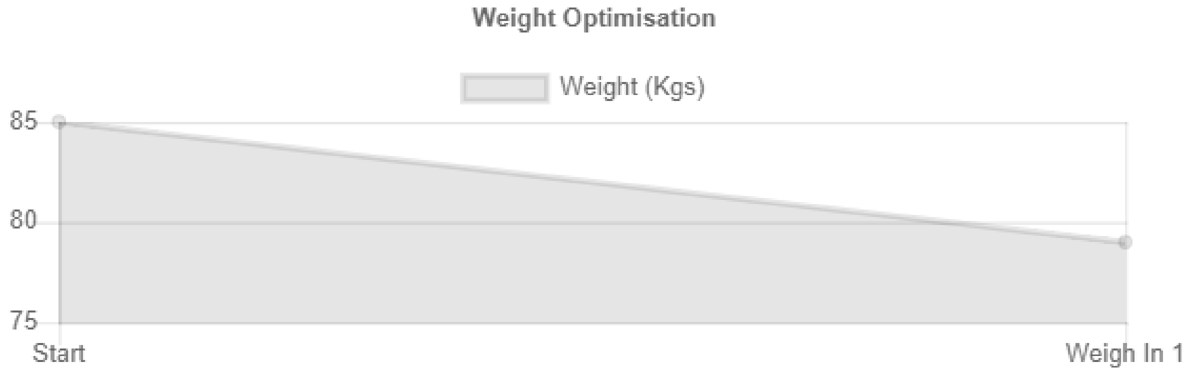

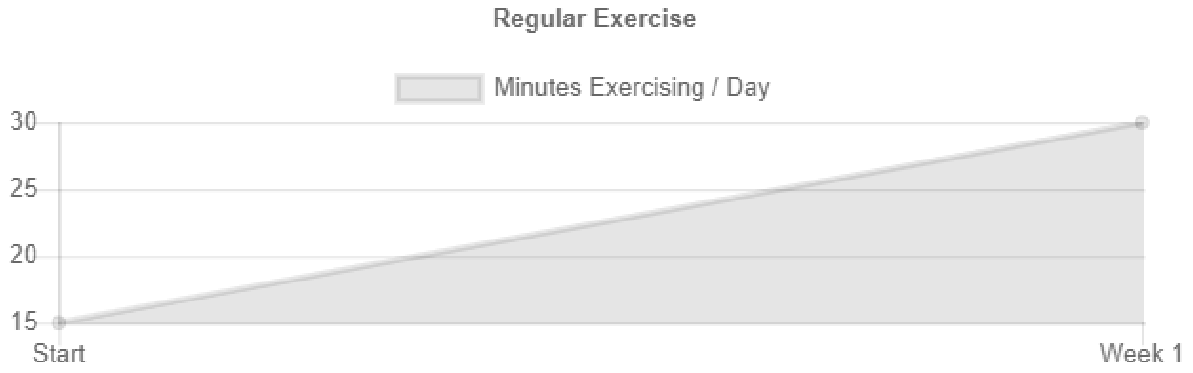

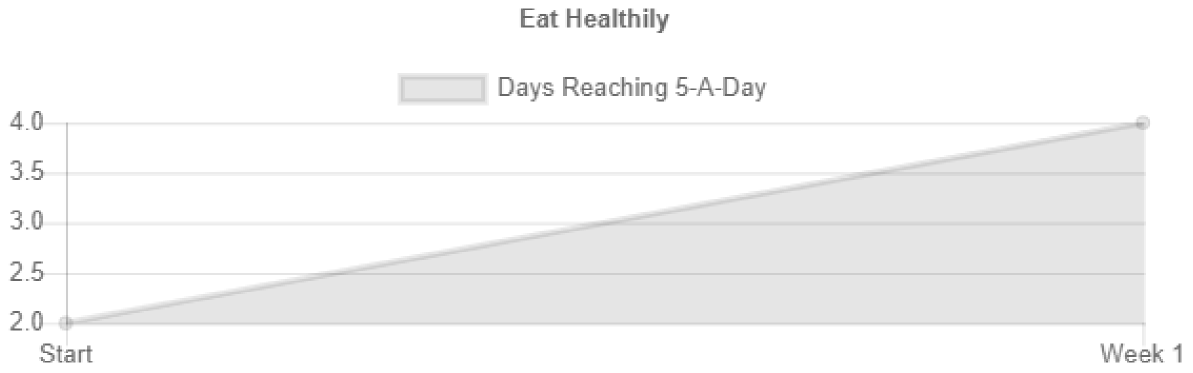

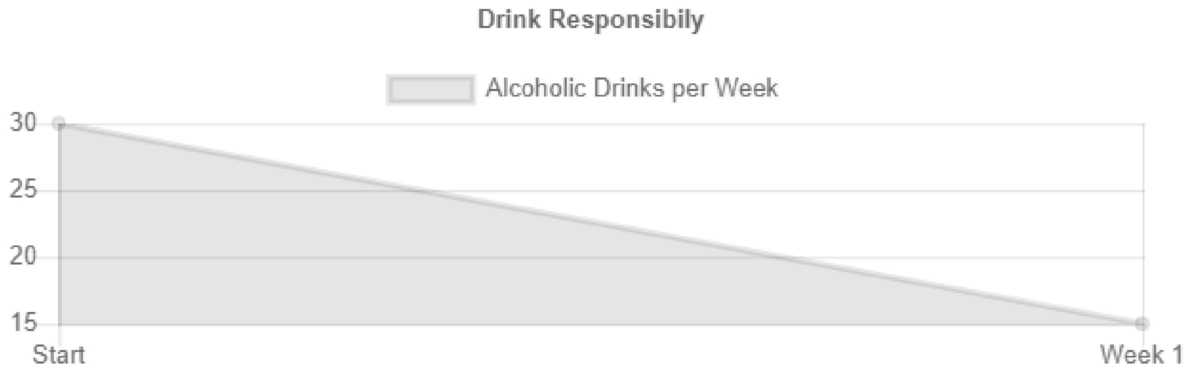

Supplement: Multimedia Appendix 3 [file formative_v4i8e18586_app3.pdf]
